# Raising Early Achievement in Math With Interactive Apps: A Randomized Control Trial

**DOI:** 10.1037/edu0000286

**Published:** 2018-06-25

**Authors:** Laura A. Outhwaite, Marc Faulder, Anthea Gulliford, Nicola J. Pitchford

**Affiliations:** 1 School of Psychology, University of Nottingham; 2 Burton Joyce Primary School, Nottingham; 3 School of Psychology, University of Nottingham

**Keywords:** math achievement, improving classroom teaching, interactive learning environments, elementary education

## Abstract

Improving provision and raising achievement in early math for young children is of national importance. Child-centered apps offer an opportunity to develop strong foundations in learning math as they deliver one-to-one instruction. Reported here is the first pupil-level randomized control trial in the United Kingdom of interactive math apps designed for early years education, with 389 children aged 4–5 years. The original and rigorous research design disentangled the impact of the math apps as a form of quality math instruction from additional exposure to math. It was predicted that using the apps would increase math achievement when implemented by teachers in addition to standard math activities (treatment) or instead of a regular small group-based math activity (time-equivalent treatment) compared with standard math practice only (control). After a 12-week intervention period, results showed significantly greater math learning gains for both forms of app implementation compared with standard math practice. The math apps supported targeted basic facts and concepts and generalized to higher-level math reasoning and problem solving skills. There were no significant differences between the 2 forms of math app implementation, suggesting the math apps can be implemented in a well-balanced curriculum. Features of the interactive apps, which are grounded in instructional psychology and combine aspects of direct instruction with play, may account for the observed learning gains. These novel results suggest that structured, content-rich, interactive apps can provide a vehicle for efficiently delivering high-quality math instruction for all pupils in a classroom context and can effectively raise achievement in early math.

## Educational Impact and Implications Statement

In a pupil-level randomized control trial we evaluated the effectiveness of a new math app intervention to support young children’s early math development. The results showed children using the math apps either as a supplementary intervention or instead of a small group teacher-led math activity made significant learning gains in comparison with children receiving standard practice only. This study suggests high-quality math apps can be used as a form of quality math instruction in a well-rounded curriculum to raise achievement in early math for all children.

Raising achievement in mathematics is an issue of national importance. In the United Kingdom (U.K.) attainment in early math has been shown to lag behind attainment in early literacy within the same group of children, revealing a significant discrepancy in the development of these two domains in the first year of formal education (Department for Education, 2016; Pitchford, Papini, Outhwaite, & Gulliford, 2016). In part, this may be because of the provision of a phonics-based literacy intervention in all elementary schools in the country (Department for Education, 2010, 2016), whereas a similar strategy for mathematics is not currently implemented. However, developing a strong foundation in early math skills is vital for children’s later educational success (Duncan et al., 2007; Geary, 2011a) and economic, health, and employment outcomes (Reyna, Nelson, Han, & Dieckmann, 2009). In response, recent research and policy calls for a greater focus on learning math at the start of school (APPG, 2014; Ofsted, 2018; Pitchford et al., 2016) through effective, efficient, and evidence-based interventions to support early math development and raise math achievement (Butterworth, Varma, & Laurillard, 2011; Jordan & Levine, 2009).

## Math Development

Math development requires the acquisition of different component skills and processes that range in level of difficulty (Goswami, 2006; Holmes & Dowker, 2013). Components of math knowledge can be grouped into four broad categories: factual knowledge (e.g., number bond combinations and properties of shape and patterns) and conceptual understanding (e.g., identifying and applying mathematical procedures), which both encompass basic math skills. In contrast, mathematical reasoning (e.g., making deductions and inferences from mathematical information), and problem solving (e.g., combining and applying different areas of mathematics to solve a problem in a specific context) reflect higher-level mathematical skills that require the application of basic math knowledge to find a solution (Rutherford-Becker & Vanderwood, 2009; Thurber, Shinn, & Smolkowski, 2002). Research shows the acquisition and automatization of basic math skills facilitates higher-level mathematical development (Codding, Archer, & Connell, 2010; Gersten & Chard, 1999; Mayfield & Chase, 2002; VanDerHeyden & Burns, 2005). For example, longitudinal research shows early factual and conceptual knowledge, including, number bond combinations, counting, pattern knowledge, and calculation ability predict more complex skills, such as problem solving later in development (Björn, Aunola, & Nurmi, 2016; Fuchs et al., 2006; Rittle-Johnson, Fyfe, Hofer, & Farran, 2016). In contrast, poor fluency in basic math skills is shown to be commonly associated with mathematical difficulties (Geary, 1993; Jordan, Hanich, & Kaplan, 2003). Together, this evidence supports theories of cumulative learning that propose the mastery of basic math facts and concepts are an essential foundation for the acquisition of more complex math skills (Gagné, 1968). It emphasizes the vital, foundational role of strong basic math skills in successful mathematical development.

However, many children struggle to acquire basic math facts and conceptual knowledge (Geary, 2011b), which makes them vulnerable to persistent underachievement throughout their education (Duncan et al., 2007; Jordan, Kaplan, Ramineni, & Locuniak, 2009). Efficiency in basic math skills can be facilitated through targeted practice, which emphasizes task repetition for effective skill acquisition (Daly, Martens, Barnett, Witt, & Olson, 2007; Imbo & Vandierendonck, 2008) and direct instruction (Chodura, Kuhn, & Holling, 2015; Kroesbergen & Van Luit, 2003; Swanson & Hoskyn, 1999), which is characterized by deliberately sequenced small units of information taught explicitly (Kirschner, Sweller, & Clark, 2006). Intervention studies show that individualized training that places the child at the center of their learning with learning activities that incorporate targeted practice and direct instruction can enhance the development of targeted basic math knowledge and generalize to other more complex math components not included in the intervention (Fuchs et al., 2009; Kidd et al., 2013; van der Ven, Segers, Takashima, & Verhoeven, 2017). This evidence provides further support for theories of cumulative learning (Gagné, 1968) and the importance of developing a strong foundation in basic math skills. It also suggests well-designed individualized early interventions that include targeted practice and direct instruction are needed to provide all children with the necessary learning opportunities to develop a strong foundation in math (Stacy, Cartwright, Arwood, Canfield, & Kloos, 2017). Such approaches may be particularly beneficial in the first years of schooling (Clements, Baroody, & Samara, 2013) when children show the fastest rates of math development (Hill, Bloom, Black, & Lipsey, 2008).

## App Technology

Educational math apps delivered on touch-screen tablets offer an opportunity for individualized math practice targeted to children’s needs. Apps that are grounded in learning science theory (Hirsh-Pasek et al., 2015) and incorporate the principles of universal design and play (Burgstahler, 2012) can provide a blended learning approach (Naismith, Lonsdale, Vavoula, & Sharples, 2004). Specifically, apps that embody the principles of active, engaged, meaningful, and socially interactive learning with a specific learning goal (Hirsh-Pasek et al., 2015) can combine benefits of direct instruction (Kirschner et al., 2006), for example, feedback, repetition, and rewards with features of free play (Gray, 2015) particularly, self-regulation and control. This can help provide an efficient child-centered but scaffolded learning environment (Mayer, 2004; Mayo, 2009) tailored to individual needs (Slavin & Lake, 2008) enabling individualized and structured instruction (Gulliford & Miller, 2015) without additional, time-consuming, teaching demands (Hilton, 2016; Kucian et al., 2011).

Educational apps delivered on touch-screen tablets are also particularly suited for young children, because they typically find them motivating (Flewitt, Messer, & Kucirkova, 2015) and intuitive to use (Cooper, 2005). Touch-screen tablets are mobile, light weight, and do not rely on dexterity-based motor skills that are needed to use a computer keyboard or mouse (Kucirkova, 2014). Furthermore, access to mobile devices in educational settings is increasing. For example, in the United Kingdom, 70% of elementary schools have access to touch-screen tablets (Clarke, 2014).

## Previous Research

Despite the prevalence, popularity, and potential benefits of using app technology to support math development, the current evidence-base is fragmented (Haßler, Major, & Hennessy, 2015) and suffers from a paucity of rigorous scientific investigations (Cheung & Slavin, 2013). Concerns have also been raised about the impact of technology based screen time on early child learning and development (Greenfield, 2015; Palmer, 2007; Sigman, 2012). To evaluate the impact of app technology in educational settings, practical and high-quality research is needed (Cheung & Slavin, 2013) and should focus on the quality of the educational app content (Blum-Ross & Livingstone, 2016; Falloon, 2013).

Emerging experimental evidence demonstrates the effectiveness of different high-quality math apps with early years pupils in a classroom setting (Outhwaite, Gulliford, & Pitchford, 2017; Pitchford, 2015; Schacter & Jo, 2016, 2017; van der Ven et al., 2017) and increasing time spent on learning math through using educational apps at home positively benefits children’s achievement in school (Berkowitz et al., 2015). All of the math apps evaluated in these studies were grounded in evidence based learning theory, embodying the principles of active, engaged, meaningful, and socially interactive learning with a specific learning goal (Hirsh-Pasek et al., 2015). Common features in these apps include explicit instruction, repetitive and cumulative training in mathematical concepts, immediate feedback, challenge and early reward, and individualized, self-paced learning, which are important components of effective math interventions (Baker, Gersten, & Lee, 2002; Fuchs et al., 2008; Gersten et al., 2009).

## Current Study

### Math App Intervention

The math apps at the focus of this study also include many of these features. Active learning in the math apps is fostered through the direct manipulation of virtual objects, verbal labels, and numerical representations (Lindahl & Folkesson, 2012). The simultaneous presentation of auditory and visual inputs engenders multisensory learning and which has been shown to facilitate children’s understanding (Carr, 2012; Pavio, 1986). Engaged learning is supported by immediate feedback (positive or negative) given after every interaction with the apps highlighting the potential of app-based learning for motivational enhancement (Couse & Chen, 2010). Furthermore, the multitouch nature of the tablet device affords cognitive embodiment, shown to be influential in mathematical development (Calder, 2016; Duijzer, Shayan, Bakker, Van der Schaaf, & Abrahamson, 2017). Meaningful learning through the math apps is promoted through a staged curriculum that builds on the child’s previous knowledge (Magliaro, Lockee, & Burton, 2005) and extends children beyond their current ability level (Inal & Cagiltay, 2007; Vygotsky, 1978). Moreover, the math apps include continuous assessment of knowledge acquired through the different topics taught. This engenders retrieval-based learning, shown to improve learning outcomes (Dunlosky, Rawson, Marsh, Nathan, & Willingham, 2013; Grimaldi & Karpicke, 2014). When using the math apps children can regulate their pace of learning within their own in-app profile, which can provide effective scaffolding for pupils with differing needs and create an individualized learning environment (Gulliford & Miller, 2015; Slavin & Lake, 2008). This can promote learner autonomy, shown to be effective for improving educational outcomes (Morrison, Ross, & Baldwin, 1992). Socially interactive learning is evident in the math apps with the on-screen teacher providing demonstrations and task instructions, which children have the opportunity to repeat when needed (Troseth, 2010). This can offer the efficient and effective delivery of one-to-one instruction, which has been shown to be an important component of math interventions (Holmes & Dowker, 2013).

Despite recent progress in the emerging evidence base for the effectiveness of educational apps to support the acquisition of early math skills, two important questions still need to be addressed: (a) how are the math apps most effectively implemented in a classroom setting compared to standard instructional practice? (b) Which components of math development are supported by the math apps?

### Effectiveness and Implementation

First, there is a need to understand how educational apps are best implemented in a classroom setting. Typically, researchers have implemented app-based interventions (e.g., Schacter & Jo, 2017) as a supplementary teaching aid, in addition to standard math practice (e.g., Berkowitz et al., 2015). This entails greater instructional time on learning mathematics compared with the comparison groups, rendering it difficult to disentangle the effects of the intervention from the effects of extra time learning math (Foster, Anthony, Clements, Sarama, & Williams, 2016; Ginsburg & Smith, 2016). To address this threat, experimental study designs need to include a time-equivalent control group (e.g., Holmes & Dowker, 2013). Furthermore, it is critical that teachers implement the intervention, to ensure high ecological validity and to support the generalizability of the intervention beyond the research context (Clements, Sarama, Wolfe, & Spitler, 2015). To address this issue in the current study, two forms of teacher-based implementation of an educational math app intervention were compared to standard math practice. As illustrated in Table 1, children in Group 1 (treatment) used the math apps in addition to all other standard math activities and so had increased exposure to math instruction. In contrast, children in Group 2 (time-equivalent treatment) used the math apps instead of a daily small group-based math activity, so time spent learning math was equivalent to the children in Group 3 (control) receiving standard teacher-led math instruction that included a daily small group-based activity. Thus, in the current study, all children received whole class math instruction delivered by the teacher, which was embedded into play-based learning, as is standard practice for early years classrooms in the United Kingdom.[Table-anchor tbl1]

In summary, this study asked, do children make more progress when the math app intervention is implemented by teachers in addition to regular math instruction (Group 1) or when implemented instead of a daily small group-based math activity (Group 2) compared with children receiving standard instructional practice (Group 3)? Based on previous research (Outhwaite et al., 2017; Pitchford, 2015), it was predicted that children who used the math apps (Group 1 and Group 2) would progress more than children receiving standard math instructional practice (Group 3), and children who received the math apps in addition to their regular math instruction (Group 1) would have the strongest learning gains.

### Components of Math Development

Second, there is a need to examine which components of math development are supported by educational apps. Previous research evaluating app interventions has frequently used assessments closely aligned with the intervention content, which typically focuses on specific aspects of math knowledge (e.g., Schacter & Jo, 2017). Studies are required to take a broader view of mathematics and consider how educational apps support the acquisition of targeted components of math knowledge and whether this generalizes to higher-level skills. This will help elucidate how math development is supported by interactive, individualized, educational apps. To address this, the math apps evaluated in this current study primarily targeted basic math facts and concept knowledge (see Table 2) and a standardized assessment of early mathematical skills that comprised measures of the four components of early mathematical development outlined above was given to all children in the trial, before and after the intervention period. This enabled learning gains for each mathematical component, including targeted basic skills and higher-level knowledge not included in the intervention to be compared across the three intervention groups. Therefore, this study also asked, for each of the four components of math development, do children make more progress with the apps when used in addition to regular math instruction (Group 1) or when implemented instead of a daily small group-based math activity (Group 2) compared with children receiving standard instructional practice (Group 3)?[Table-anchor tbl2]

## Method

### Design

A pupil-level randomized control trial (RCT) was conducted to evaluate the effectiveness of a new math app intervention compared with standard math classroom practice with children aged 4–5 years, in the first year of compulsory education in the United Kingdom, known as Early Years Foundation Stage II. The study was conducted in the last 14 weeks of the school year, before the children transitioned to the next stage of education, known in the United Kingdom as Key Stage I. There were 12 participating schools across Nottingham and Nottinghamshire in the East Midlands, United Kingdom, representing a range of socioeconomic and multicultural backgrounds and relatively high levels of educational underachievement compared to other regions in the United Kingdom (Ofsted, 2013). Within each class of the 12 participating schools, children were randomly allocated to one of three groups outlined in Table 1. As randomization occurred at the pupil-level within each class, this controlled against school effects influencing results.

This three-Group RCT design allowed the effects of maturation (Group 3: control) to be disentangled from the effects of the math apps (Group 1 and 2) being investigated. Furthermore, the inclusion of two treatment groups enabled the effects of the math apps as a form of quality instruction (Group 2: time-equivalent treatment) to be differentiated from additional exposure to math instruction (Group 1: treatment). Thus, these two forms of app implementation addressed calls from previous research and policy (APPG, 2014; Ofsted, 2018; Pitchford et al., 2016) by considering if an increased focus on early math should take the form of additional time learning math (treatment) or if current allocated time can be used more efficiently (time-equivalent treatment).

Children were assessed on the Progress Test in Math, level 5 (PTM5; Maths Assessment Resource Service, 2015), a standardized assessment of mathematical skills, before (pretest) and immediately after (posttest) an intervention period in which teachers implemented the math apps for 30 min each day across 12 consecutive weeks. The School of Psychology Ethics Committee at the University of Nottingham granted ethical approval for the study. Opt-in informed parental consent was obtained for all participating children in line with the British Psychological Society ethical guidelines. There were 85% of all available children across the 12 participating schools given parental consent to take part in the study.

### Participants

The CONSORT (2010) data in Table 3 summarizes the study sample at each stage of the RCT. In total, 461 children aged 4–5 years were randomly allocated to one of the three groups. There were 153 children assigned to Group 1 (treatment) and received the math app intervention as well as all daily standard math practices. There were 152 children randomly allocated to Group 2 (time-equivalent treatment) and used the math app intervention instead of a daily small group-based math activity that is given as part of standard math practice. The remaining 156 children were assigned to Group 3 (control) and received regular math teaching practice only.[Table-anchor tbl3]

There were 452 children from the 12 schools pretested on the PTM5 (Math Assessment Resource Service, 2015). Nine children were absent at pretest but were still randomized to group. Of the 452 children that were pretested, 389 children from 11 schools were available at posttest and were given the same math assessment immediately after the 12-week intervention period. In total, 63 children who were pretested did not complete the posttest; one child left school, two children were removed from the study by their teachers for reasons unknown, and 60 children were absent at posttest, including 30 children from one school because of a school fieldtrip. It was not possible to follow-up on children that were absent on the day of the posttest because posttesting took place during the last week of the school year. Table 4 details descriptive data for the final sample of 389 children.[Table-anchor tbl4]

### Math App Intervention

The intervention consisted of two math apps, “Maths 3–5” and “Maths 4–6,” developed by *onebillion*, an educational not-for-profit organization (www.onebillion.org.uk). These math apps are based on core mathematical concepts in Number and Shape, and Space and Measure, covered in the Early Years Foundation Stage (EYFS) Profile (Department for Education, 2013; see Table 2). The apps also start to introduce children to topics included in the U.K. National Primary Curriculum for Key Stage I (Department for Education, 2014). The apps primarily target factual knowledge and basic conceptual understanding, for example, simple numerical operations, such as addition and subtraction. Table 2 details the topics covered in each app and how the app content maps onto the math curriculum and the components of math development.

Features of the math apps and how they map onto the principles of active, engaged, meaningful, and socially interactive learning are discussed in detail above. Overall, the apps are designed to deliver child-centered tuition through interactive picture, audio, and animation formats with clear objectives, instructions, and immediate formative feedback, consistent for all users. Children work through the apps individually with headphones, at their own pace, and have the opportunity to repeat instructions and activities as often as needed. To complete a topic, children need to achieve 100% pass rate on an end of topic quiz included in the software. The quizzes are designed to assess children’s knowledge of the mathematical concepts covered in the topic activities.

**Figure 1 fig1:**
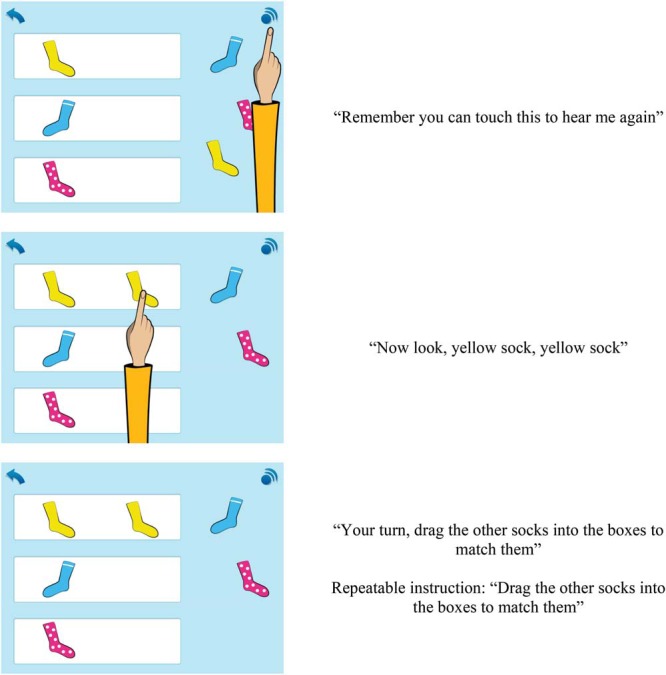
Example item and task instructions for Topic 1, Sorting and Matching.

For example, in topic 1 in Maths 3–5 children are taught the concepts of sorting and matching through a range of activities involving sorting and matching different items by type, shape, size, and color. Screenshots of example activity items and task instructions for Topic 1 are displayed in Figure 1 (courtesy of *onebillion*). After completing seven sets of activities, children reach the end of topic quiz that includes 10 questions from the previous activities. When children pass the quiz they are awarded a certificate and progress to the next topic.[Fig-anchor fig1]

As shown in Table 1 children in Group 1 (treatment) and Group 2 (time-equivalent treatment) received the daily math app intervention for 30 min each day over the 12-week intervention period.

### Small Group Math Instruction

Small group math instruction was consistent with the Number and Shape, and Space and Measure content covered in the EYFS Profile (Department for Education, 2013; see Table 2). Group-based activities were delivered by the class teacher and focused on a particular mathematical concept from the EYFS Profile. Example activities were obtained through observations made during school visits by the first author. For example, shape recognition was taught by the teacher drawing different shapes on the whiteboard and asking the small group of children, “what shape am I?” Children responded by calling out the answer and receiving corrective feedback from the teacher before moving onto the next item. In an activity focused on understanding the concepts of more and less, the teacher utilized a number line visual aid and physically demonstrated “1 more than 18.” The teacher then asked the small group of children, “what is 1 more than 10?” and children responded by writing their answer on an individual mini whiteboard and showing it to the teacher. The teacher would then give corrective feedback before moving onto the next item. As highlighted in Table 1, children in Group 1 (treatment) and children in Group 3 (control) received instruction through daily small group math activities as part of standard math practice.

### Whole Class Embedded Math Activities

In the United Kingdom children in the first year of school (aged 4–5 years old) are typically taught through play. In a whole class setting teachers embed mathematical concepts from the EYFS Profile into play-based activities. Examples of whole-class activities were obtained through observations made during school visits by the first author. For example, children were introduced to the concept of volume in a water play activity with containers of different sizes placed in the wet play area. During times of free play children had the opportunity to explore which of the different containers could hold the most water. The concept of shape recognition was introduced to children by identifying shapes in their environment. In this activity children explored their classroom environment to find examples of different shapes, which were recorded on a teacher-made worksheet. Teaching staff supervised both activities to facilitate and scaffold children’s exploratory play. As shown in Table 1 all children participating in this study received this form of teacher-led embedded instruction.

### Math Assessment

Children’s mathematical ability was assessed using the PTM5 (Math Assessment Resource Service, 2015). The PTM5 is a paper-based, age-appropriate, standardized measure of mathematical ability, applicable for children aged 4–5 years old in the summer term of the Early Years Foundation Stage II, when this trial took place. The assessment is designed to be used by schools to track children’s progress and has been used in other evaluation studies of educational interventions (Jerrim & Vignoles, 2016; Worth, Sizmur, Ager, & Styles, 2015). It covers a concise selection of items from the EYFS Profile (Department for Education, 2013; see Table 2) and is independent of the math app intervention. The PTM5 assessment can be delivered to groups of pupils, but for this study the assessment was administered on a one-to-one basis, to maximize child engagement. Reliability analysis of the PTM5 assessment using a one-to-one administration procedure showed high internal consistency between pretest and posttest scores, *r* = .67, Cronbach’s α = .80.

The PTM5 is designed to assess the four components of math proficiency outlined in the introduction, with increasing levels of difficulty and application of mathematical knowledge, for example: (a) Fluency in Facts and Procedures using ordinal numbers and recognizing shapes (maximum raw score = 7); (b) Fluency in Conceptual Understanding measures understanding “most” and “least” and recognizing numbers of quantity (maximum raw score = 9); (c) Mathematical Reasoning assesses drawing conclusions from mathematical information (maximum raw score = 7); and (4) Problem Solving assesses making connections between different parts of math to solve a problem in a particular context (maximum raw score = 3). The total maximum raw score was 26 with no discontinuation rule; children completed all questions on this assessment.

### Procedure

#### School recruitment

A recruitment event was held through the Nottingham Apple Education Regional Training Centre to inform interested schools about the study and the math app intervention being trialed. Participating schools already had access to the required hand-held tablet device hardware (iPads). Participating schools were given access to the math apps, free of charge, by *onebillion*. Children were recruited through schools that agreed to participate in the study and opt-in parental consent was obtained for all participating children before study commencement.

#### Implementation monitoring

A teacher manual was produced to provide further guidance on how to implement the math app intervention and included full details of the study protocol to maximize consistency across all participating schools. The manual did not provide instructions for when and how mathematics should be taught in standard practice. The implementation of standard practice was consistent with national guidelines provided in the EYFS Profile (Department for Education, 2013; see Table 2) and was at the discretion and autonomy of the teaching and senior staff, as is standard in England (The EYFS Profile is not statutory in Scotland and Wales). The first author also visited each school before the trial commenced to ensure the teaching staff were fully informed of the study protocol and had successfully embedded the math app intervention within their daily school routine. Although, school implementation timetables varied because of iPad availability and individual school routines, the first author visited all schools again during the 12- week intervention period to interview the teaching staff about their experiences of the math apps, observe the math app intervention sessions and ensure intervention compliance. The small group math instruction and whole class embedded math activities were also observed to gain an understanding of standard math practice, however, systematic monitoring of implementation fidelity was not practically possible in the current study.

#### Group allocation

Within participating schools, children in each class were randomly assigned to one of the three groups (see Table 1). This controlled for potential school and teacher effects, as the apps were implemented by the usual teaching staff in the 12 participating schools to optimize ecological validity. As each participating class typically had one teacher and one or two teaching assistants this allowed all children from each class to be taught/supervised by their usual teaching staff that were known to them. To guard against selection bias, each child was given a unique study identification number by class teachers as parental consent was returned, and these were given to the first author who then randomized children to group allocation using a random number generator. This way, the researchers were blind to group allocation. Once group allocation was complete, the first author returned a list of study identification numbers for each group to each of the class teachers taking part in the trial, who were then able to match the study identification number and group allocation to individual children taking part in the study. The group allocations remained fixed throughout the study and the researchers remained blind to the correspondence between study identification number and group allocation throughout.

#### Assessment administration

The standardized paper-based math assessment (PTM5) was administered immediately before (1 week) and immediately after (1 week) the 12-week intervention period. A team of trained assessors delivered the PTM5 to individual children, on a one-to-one basis, in a quiet area, free from distraction, in the child’s familiar school environment. Before the assessment began, the trained assessor explained the task to the child, ensuring the child felt comfortable and explicitly asked if the child would like to take part in the assessment so as to gain assent. Because of the child’s age, verbal or nonverbal assent (using a smiley face/not smiley face response sheet) was accepted. The trained assessor then read aloud the questions from the PTM5, one at a time, and the child was required to write their response in the response booklet. Each math assessment lasted 10–15 min per child. The trained assessors were recruited from the University of Nottingham and were trained on administration by the first and last author. All trained assessors were blind to group allocation so were unaware which children had received the math app intervention. GL Assessment independently scored all of the completed assessments and was also blind to condition.

#### Intervention implementation

Children assigned to receive the math app intervention in Group 1 (treatment) and Group 2 (time-equivalent treatment) used the apps for approximately 30 min a day for 12 weeks. Children used the same iPad each day, as they had their own profile in the apps, which saved their progress. To support classroom organization, iPads were color coded or numbered by the teaching staff. The intervention was administered in small groups of between 10 and 15 children, depending on class size. Children worked independently through the apps, using headphones in a quiet area of the classroom, supervised by teaching staff that provided technical support and ensured children remained focused on the tasks.

The intervention was embedded into the daily classroom routine. As highlighted in Table 1, children assigned to Group 2 (time-equivalent treatment) used the intervention while the other children received a small group-based math input activity. This ensured children in Group 2 (time-equivalent treatment) received the same amount of time on math education as Group 3 (control). Children assigned to Group 1 (treatment) used the intervention at a different time during the school day, for example, during free play sessions, so as not to miss out on core subjects, such as phonics. Despite being implemented during free play, children were supervised and instructed to use the math apps by teaching staff; it was not an optional activity, as is sometimes conventional in free play. As such, children in Group 1 (treatment) had more exposure to math instruction over the 12-week intervention period. At the end of the study, the participating schools continued to have access to the math apps, so the apps were available to all pupils.

## Results

### Preliminary Analyses

Standardized norms for the PTM5 are based on group administration (Maths Assessment Resource Service, 2015) but as this study used one-to-one administration to maximize child engagement standardized scores were not considered appropriate. Instead, raw scores on the PTM5 were used as the dependent variable. A one-way analysis of variance (ANOVA) showed no significant age differences across the three instruction groups, *F*(2, 386) = 1.03, *p* = 0.358, confirming it suitable to use raw scores. There were also no observed gender differences across the three groups, χ(2) = 2.25, *p* = .324. Despite making directional hypotheses that the math apps would be more effective in supporting the development of early mathematical skills than standard classroom practice, all analyses are reported at a two-tailed level of probability, unless otherwise stated.

### Effectiveness and Implementation

To establish which form of implementation of the math app intervention was the most effective compared to standard classroom practice in supporting the acquisition of early math skills and to account for minor differences in pretest math ability across the three groups (see Table 5; Van Breukelen, 2006), mean math performance for each of the three groups (see Table 5) was compared using a 2 (Time: pretest, posttest) × 3 (Group: Group 1, Group 2, Group 3) mixed ANOVA. Results showed a significant interaction between Time and Group, *F*(2, 386) = 3.10, *p* = 0.046.[Table-anchor tbl5]

Analysis of simple main effects showed no significant differences between groups at pretest, *F*(2, 386) = 1.63, *p* = 0.197, or posttest, *F*(2, 386) = 0.12, *p* = 0.888. Over time, significant learning gains were found for Group 1 (treatment), *t*(125) = 10.54, *p* < .0001, Group 2 (time-equivalent treatment), *t*(130) = 8.97, *p* < .0001, and Group 3 (control), *t*(131) = 7.03, *p* < .0001. The largest within-group effect sizes (Cohen’s *d* with 95% confidence interval [CI]) reflecting the magnitude of progress were observed for Group 1 (treatment; see Table 5).

To explore the significant interaction further, planned comparisons with independent samples *t* tests and between-groups effect sizes (Cohen’s *d* with 95% CI; Trafimow, 2015) were conducted on progress made over time (difference scores; posttest minus pretest). Results showed that pupils in Group 1 (treatment) made significantly more progress over the 12-week intervention period than Group 3 (control), *t*(256) = 2.46, *p* = 0.015, between-groups effect size = 0.31, CI = 0.06–0.55. Similarly, pupils in Group 2 (time-equivalent treatment) made significantly greater learning gains compared with pupils in Group 3 (control) in line with the predictions, *t*(261) = 1.74, *p* = 0.042, one-tailed, between-groups effect size = 0.21, CI = −0.03–0.46. There was no significant difference in the progress made by Group 1 (treatment) and Group 2 (time-equivalent treatment), *t*(255) = 0.64, *p* = 0.525, between groups effect size = 0.08, CI = −0.17–0.33.

### Components of Mathematical Proficiency

To examine how effective the math apps were at supporting the acquisition of the four components of mathematical proficiency (Fluency in Facts, Fluency in Concepts, Mathematical Reasoning and Problem Solving) compared with normal classroom practice, mean performance on each component for the three groups (see Table 5) were compared using separate 2 (Time: pretest, posttest) × 3 (Group: Group 1, Group 2, Group 3) mixed ANOVAs. Results showed no significant Time × Group interactions for any of the four components; Fluency in Facts *F*(2, 386) = 0.56, *p* = 0.570; Fluency in Concepts *F*(2, 386) = 2.04, *p* = 0.132; Mathematical Reasoning *F*(2, 386) = 1.37, *p* = 0.256; Problem Solving *F*(2, 386) = 1.42, *p* = 0.244.

## Discussion

This study reports the first pupil-level RCT to be conducted evaluating the effectiveness of a new math app intervention for children aged 4–5 years old in the United Kingdom. Specifically, this RCT evaluated two forms of math app intervention implementation (treatment and time-equivalent treatment) compared with a standard practice control. This is the first math app intervention study to adopt this novel and rigorous research design. The current findings are of particular significance to the provision of early math instruction and the need to raise math achievement in the early years of education (APPG, 2014; Ofsted, 2018; Pitchford et al., 2016).

### Math App Intervention Effectiveness and Implementation

This study found that combining child-centered, curriculum-based, apps with interactive touch-screen tablet technology for children aged 4–5 years old in the first year of school provides an effective means of delivering quality instruction that promotes the development of early math skills. Specifically, the interpretation of between-groups effect sizes based on normative expectations of change (Hill et al., 2008) showed at the whole sample level, children in Group 1 (treatment) who used the math apps in addition to all normal math practices were 3–4 months ahead of their peers in Group 3 (control) receiving standard practice only (between-groups effect size 0.31). Children in Group 2 (time-equivalent treatment) who used the math apps instead of one daily regular small group math activity were shown to be approximately 2 months ahead of children in Group 3 (control) receiving standard practice only (between-groups effect size 0.21). However, there was no significant difference between implementing the math apps as well as all standard math practices or instead of one regular small group math activity. This indicates the apps are a form of quality math instruction and suggests an increased focus on early math at the class level (APPG, 2014; Ofsted, 2018; Pitchford et al., 2016) can take the form of efficient instructional practices without the need for extra time learning math, which could potentially detract from other areas of a well-rounded curriculum.

### Supporting Components of Math Development

When examining which components of math development are supported by the math app intervention, results showed no significant Time × Group interactions for any of the four components of mathematical proficiency. This suggests all three forms of instruction supported the four areas of math development. However, when comparing within-group effect sizes (see Table 5), which reflect the magnitude of progress in each group, larger effect sizes were consistently observed for children who used the math apps (Group 1 and Group 2) compared with children who received standard practice (Group 3) across all four math components. This suggests the math apps strongly support learning in the areas of math development targeted by the intervention (Fluency in Facts and Fluency in Concepts; see Table 2) that generalizes to other higher-level skills not extensively or explicitly covered in the app content (Mathematical Reasoning and Problem Solving). These results suggest the math apps go beyond drill-based practice for fact retrieval (Salminen, Koponen, Leskinen, Poikkeus, & Aro, 2015) and help build a strong foundation in basic math skills that enables math facts and simple concepts to become automated and assimilated into a higher-level conceptualization of math knowledge (Codding et al., 2010; Gersten & Chard, 1999; Karmiloff-Smith, 1994; Mayfield & Chase, 2002; VanDerHeyden & Burns, 2005). These results are consistent with cumulative learning theory (Gagné, 1968) and agree with previous intervention studies that have also targeted basic math skill acquisition and demonstrated evidence of generalization to higher-level mathematical skills (Fuchs et al., 2009; Kidd et al., 2013; van der Ven et al., 2017).

### Theoretical Implications

Overall, these results corroborate previous research demonstrating proof of concept of this new math app intervention over a series of small scale studies (Outhwaite et al., 2017) and adds to the growing evidence base demonstrating the educational benefits of app content for young children (Berkowitz et al., 2015; Schacter & Jo, 2016, 2017; van der Ven et al., 2017) in different educational contexts (Pitchford, 2015). Several features of the math apps may count for their success. In particular, the math apps include features consistent with the principles of active (e.g., multisensory and direct interactions), engaged (e.g., feedback), meaningful (e.g., a staged and scaffolded curriculum), and socially interactive learning (e.g., through the on-screen teacher) as discussed earlier (Hirsh-Pasek et al., 2015). The math apps also include high-quality curriculum based content (see Table 2; Blum-Ross & Livingstone, 2016; Falloon, 2013) and specific learning goals (Hirsh-Pasek et al., 2015). Finally, the math apps draw on different instructional psychology principles, namely direct instruction through feedback, repetition, and reward (Kirschner et al., 2006) and free play through the opportunity for self-regulation and learner control (Gray, 2015). The combination of these app design features may account for the observed learning gains.

An interesting find was that the math app learning gains found in this study conducted in a high-income Western country (within-group effect size >0.65) were comparable with the learning gains (within-subject effect size >0.80) observed in Pitchford’s (2015) study evaluating the same math apps in Malawi, a low-income country with a history of poor child development (Hubber et al., 2016) and extremely low basic math skills (UNESCO-IBE, 2010). Children in Malawi used the same math apps, with instructions delivered in their local language. Although interventions are not typically considered universally effective (Fuchs et al., 2009), this study shows comparable learning gains with the same math apps in two radically different educational contexts, suggesting these app design principles based on science of learning theory may be a common mechanism underpinning learning with high-quality educational app content that transcends culture.

### Policy and Practice Implications

The significance of the study results also has two important implications for policy and practice. First, previous intervention research has predominately focused on low-achievers and with small sample sizes (e.g., Outhwaite et al., 2017; Räsänen, Salminen, Wilson, Aunio, & Dehaene, 2009; Schacter & Jo, 2016; Wilson, Revkin, Cohen, Cohen, & Dehaene, 2006). In contrast, this study implemented the math app intervention at the whole class level with a final sample of 389 children. This novel approach is important as typically attaining and low-achieving pupils both demonstrate difficulties acquiring early mathematical skills (Dowker, 2005; Geary & Hoard, 2005) so high-quality app based math instruction may be suitable for all children in early education. Furthermore, it clearly addresses previous research and policy calling for instructional practices that benefit the math development of all children (APPG, 2014; Ofsted, 2018; Pitchford et al., 2016) to address the “math-practice” gap (Stacy et al., 2017) and close the gap in math and literacy attainment in the first year of school (Pitchford et al., 2016).

Second, previous research has typically evaluated math app interventions as a supplementary teaching aid (e.g., Berkowitz et al., 2015; Outhwaite et al., 2017). The application of a three-arm RCT in this study with two forms of intervention implementation (treatment and time-equivalent treatment) compared with a standard practice control enabled the impact of the math apps to be disentangled from additional exposure to math instruction (Holmes & Dowker, 2013). This novel and rigorous research design is particularly beneficial to address concerns raised in response to recent U.K. based early years educational practice recommendations (Ofsted, 2018). Critiques of educational policy have argued that the early years curriculum introduces too much time spent on formal instruction, reducing opportunities for play-based exploratory learning (Roberts-Holmes, 2015). This study shows the math apps were an efficient form of math instruction and shows the apps can be implemented as part of a well-balanced curriculum. It indicates the apps can capitalize on the benefits of play based learning by introducing children to math concepts in a game like format, that is interactive, engaging and child centered, but also draws on the benefits of direct instruction and targeted practice, helping to bridge the gap between formal and informal early math learning opportunities (Evangelou, Sylva, Kyriacou, Wild, & Glenny, 2009).

### Limitations and Future Directions

The current study adds to the mounting evidence base evaluating this new math app intervention developed by *onebillion* as part of a staged scaling of implementation (Outhwaite et al., 2017; Pitchford, 2015). Blind group allocation, the inclusion of a time-equivalent treatment group and an independent standardized math assessment help to address potential threats to validity and current concerns regarding the usefulness of RCTs in educational research (Ginsburg & Smith, 2016). However, there are three important limitations to consider when interpreting the results of the current study and directing future research. Further lines of research and implications for classroom practice are also discussed.

First, there were minor differences in the instructional content between the intervention treatment groups and the control group. Specifically, the math apps included a number of topics from the U.K. National Primary Curriculum for Key Stage I (see Table 2). This poses a potential threat to validity (Cheung & Slavin, 2013; Ginsburg & Smith, 2016; Shadish, Cook, & Campbell, 2002) as children in the intervention groups had access to more advanced topics than children in the control group. However, the independent assessment measure only included a concise selection of items from the EYFS Profile (Department for Education, 2013; see Table 2). As children were not assessed on the more advanced topics from Key Stage I, for example, odd and even numbers, the intervention groups did not have an unfair advantage over their peers in the control group in the assessment procedure.

Second, as the intervention was implemented toward the end of the first year of school, some of the higher ability children in the two intervention treatment groups may have already mastered some of the math skills covered in topics that are presented early in the apps. The revision of math content may have benefitted their learning (Dunlosky et al., 2013) and boosted their confidence with the app technology. Alternatively, it may have delayed further progress within the 12-week intervention period. In contrast, children in the standard practice control group continued to work through the early years curriculum content, without this revision. To address this issue, entry-level placement strategies should be developed consistent with the curriculum and components of math development (Kadosh, Dowker, Heine, Kaufmann, & Kucian, 2013) and results from future research utilizing this tool should be compared to this study to examine the impact of revision on math progress.

Third, while this study indicates the benefits of this new math app intervention for supporting the development of young children’s mathematics skills, it is important to recognize that technology alone will not lead to success; but is dependent on how the technology is integrated into the school environment (Beach & O’Brien, 2015; Couse & Chen, 2010). Detailed qualitative research is needed, therefore, to explore insights into teachers’ perceptions and implementation of using the math apps in their classrooms. Although no main effect or interactions were found in this study when School was entered as an independent variable in the analyses reported above, understanding which school level factors may impact the success of scaling this intervention will provide important insights for recommendations for best practice in implementation. This will help drive theoretical understandings of learning with educational apps and consequently help to optimize learning outcomes for all.

Finally, this study examined the effectiveness of the math app intervention for children of all ability levels. This was in response to previous research revealing a significant discrepancy in math development in the first year of formal education and recent policy calling for an increased focus on math in the early years for all children (APPG, 2014; Department for Education, 2016; Ofsted, 2018; Pitchford et al., 2016). However, when considering the usability of the math app intervention outside of the research context, many of the participating schools have since chosen to implement the math apps as a targeted, supplementary intervention for children struggling to acquire basic math skills. This is particularly interesting as children in this study who were statistically identified as low-achievers based on their pretest math score (≥1.5 *SD* below the overall group mean; Snowling, 2013) showed much stronger learning gains (within-group effect size 4.03, 95% CI = 1.87–6.18) when they used the math apps as well as all standard math practice (Group 1 treatment) relative to low-achieving peers who received standard practice only (Group 3 control, within-group effect size 1.25, 95% CI = −0.10–2.61). This observation raises the possibility that the math app intervention might be particularly beneficial for low-achieving young children who may require supplementary math instruction. However, these observations are based on a small sample of low-achievers (*n* = 31 across all three arms of the RCT) and the threats to internal validity associated with identifying low-achievers on baseline performance (Barnett, van der Pols, & Dobson, 2004) render the security of this observation very weak. Nevertheless, it is indicative for a current large scale cluster RCT evaluating these math apps as a targeted intervention for low-achieving children (identified through teacher assessments) aged 5–6 years old across 114 schools in the United Kingdom. This current efficacy trial is being delivered by the University of Nottingham and evaluated by the University of Oxford as part of an Education Endowment Foundation funded trial. The results of this large-scale trial are expected in autumn 2019.

## Conclusion

Overall, this study shows the new math app intervention was an efficient form of early math instruction for children aged 4–5 years old in the first year of education in the United Kingdom. The results support the assertion that well-designed and theoretically grounded app content can be effectively integrated into the early years classroom to deliver efficient and effective math instruction (Hirsh-Pasek et al., 2015; Kucirkova, 2014) for children of all ability levels and without placing additional time-consuming demands on teaching staff (Hilton, 2016; Kucian et al., 2011). Furthermore, through enhancing the development of basic math facts and concepts with targeted practice and direct instruction, the development of higher-level math abilities, including mathematical reasoning and problem solving, can also be supported.

These findings have important implications for addressing recent research and policy highlighting the need to raise early math attainment (APPG, 2014; Ofsted, 2018; Pitchford et al., 2016) as part of a well-balanced early years curriculum.

## Figures and Tables

**Table 1 tbl1:** Description of Math Intervention Received and Total Math-Learning Exposure for Each Group Across the 12-Week Intervention Period

Math activities	Group 1 (treatment)	Group 2 (time-equivalent treatment)	Group 3 (control)
Math app	✓	✓	
Small group-based math instruction	✓		✓
Whole class embedded math activities	✓	✓	✓
Total time learning math	Additional	Typical	Typical

**Table 2 tbl2:** Math Apps Content Mapped to the EYFS Profile Math Curriculum, Components of Math Development and Assessment Measure

App topic	EYFS profile math curriculum	Math development	Assessed in PTM5
Maths 3–5			
Sorting and matching	Shape, space, and measure	Factual knowledge	✓
	• Interest in shape and space by playing with shapes and making arrangements with objects		
	• Awareness of similarities in shape		
Counting to 3	Number	Factual knowledge	✓
	• Uses some number names		
	• Recognize and recite numbers in order		
	• Count objects that cannot be moved		
Lines and patterns	Shape, space, and measure	Factual knowledge	✓
	• Uses objects and shapes to create and recreate patterns and build models		
Counting 4 to 6	Number	Factual knowledge	✓
	• Begin to represent numbers with external objects		
	• Select correct numerals 1 to 5		
	• Count up to 6 objects using correct number name for each item		
Where is it?	Shape, space, and measure	Factual knowledge	✓
	• Uses relative positional language; e.g., behind/next to		
Counting 7 to 10	Number	Factual knowledge	✓
	• Identifying numbers in a set		
	• Match number and quantity		
Patterns and shape	Shape, space, and measure	Factual knowledge	✓
	• Select a named shape		
Counting 1 to 10	Number	Factual knowledge	✓
	• Recite numbers, select correct numerals and count objects 1 to 10 in regular and irregular arrangements		
	• One more and one less than a given number up to 10		
	• Compare two groups that have the same number		
Comparing	Shape, space, and measure	Conceptual understanding	✓
	• Order items by length, height, weight, and capacity		
	• Use “more” and “fewer” to compare two sets of objects		
Adding and taking away	Number	Conceptual understanding	✓
	• Interest in number problems		
	• Find total number of items in two groups by counting		
	• Addition and subtraction vocabulary		
Maths 4–6 (*Key Stage 1 topics)			
Shape and position	Shape, space, and measure	Factual knowledge	—
	• Describe position and distance		
	• Identify line of symmetry in shapes*		
Counting to 20	Number	Factual knowledge	—
	• Count reliably 1 to 20		
Sharing	Number	Conceptual understanding	—
	• Separate a group of objects in different ways and recognize the total is the same		
More counting	Number	Factual knowledge	—
	• Place numbers in order		
	• Count in multiples of 2*		
	• Odd and even numbers*		
Telling the time	Shape, space, and measure	Mathematical reasoning	✓
	• Use everyday language related to time		
	• Measure short periods of time in simple ways		
	• Order and sequence familiar events		
Add and subtract	Number	Conceptual knowledge	✓
	• Add and subtract two single digit numbers		
Count in 10 s and 5 s	Number	Factual knowledge	—
	• Count in multiples of 5 s and 10 s*		
How tall, how long?	Shape, space, and measure	Conceptual understanding	✓
	• Recognize shapes and objects as “tall”		
	• Compare length and height		
Count to 100	Number	Factual knowledge	—
	• Count to 100*		
Two-dimensional shapes	Shape, space, and measure	Factual knowledge	—
	• Use mathematical names to describe two-dimensional shapes		
Number lines	Number	Factual knowledge	—
	• Count reliably 1 to 20		
	• One more and one less than a given number up to 20		
Fractions	Number	Conceptual understanding	—
	• Recognize, find and name fractions of object, space, or quantity*		
*Note*. EYFS = Early Years Foundation Stage; PTM5 = Progress Test in Math, level 5; Asterisk refers to Key Stage 1 topics.			

**Table 3 tbl3:** CONSORT Table Describing the Composition of the Study Sample at Each Stage of the RCT

Stage of RCT	Total	Group 1 (treatment)	Group 2 (time-equivalent treatment)	Group 3 (control)
Randomized to group	461	153	152	156
Pretested	452	149	150	153
Absent at pretest	9	4	2	3
Withdrew during study	3	0	1	2
Left school	1	0	0	1
Removed from study	2	0	1	1
Posttested	389	126	131	132
Absent at posttest	63	23	19	21
Final sample	389	126	131	132
*Note*. RCT = randomized control trial.				

**Table 4 tbl4:** Descriptive Data, Including Mean Age in Months (SD, Min-Max) and Gender (Female: Male) for the Final Sample in the Trial

Descriptive data	Total	Group 1 (treatment)	Group 2 (time-equivalent treatment)	Group 3 (control)
Age (months)	60.64 (3.62)	60.39 (3.74)	61.00 (3.61)	60.52 (3.52)
	53.00–66.00	54.00–66.00	55.00–66.00	53.00–66.00
Gender (F:M)	196:193	60:66	73:58	63:69

**Table 5 tbl5:** Raw Score Group Mean (SD) at Pretest and Posttest With Group Mean (SD) Difference Scores (Posttest Minus Pretest), Percentage Gains, and Within-Group Cohen’s d Effect Sizes (95% CI, Confidence Interval) for the Assessment of Mathematical Ability (Max Score 26) and Each Component of Mathematical Proficiency

Math performance	Group 1 (treatment)	Group 2 (time-equivalent treatment)	Group 3 (control)
Total raw score (max. 26)			
Pretest Mean (*SD*)	11.82 (4.73)	11.85 (4.74)	12.77 (4.99)
Posttest Mean (*SD*)	15.29 (4.16)	15.02 (5.08)	15.09 (4.82)
Gain score Mean (*SD*)	3.48 (3.70)	3.17 (4.04)	2.33 (3.80)
% Gain	13.4	12.2	9.0
Cohen’s *d* (95% CI)	.78 (.42–1.14)	.65 (.29–1.00)	.47 (.13–.82)
Fluency in facts (max. 7)			
Pretest Mean (*SD*)	2.69 (1.85)	2.66 (1.78)	2.88 (1.92)
Posttest Mean (*SD*)	3.56 (1.82)	3.68 (2.06)	3.64 (1.95)
Gain score Mean (*SD*)	.87 (1.98)	1.02 (2.00)	.76 (1.93)
% Gain	12.4	14.6	10.9
Cohen’s *d* (95% CI)	.47 (.12–.83)	.53 (.18–.88)	.39 (.05–.74)
Fluency in concepts (max. 9)			
Pretest Mean (*SD*)	5.70 (2.24)	5.80 (2.18)	6.05 (2.06)
Posttest Mean (*SD*)	6.90 (1.61)	6.78 (1.81)	6.76 (1.81)
Gain score Mean (*SD*)	1.20 (2.03)	.98 (2.05)	.71 (1.72)
% Gain	13.3	10.9	7.9
Cohen’s *d* (95% CI)	.62 (.26–.97)	.49 (.14–.84)	.37 (.02–.71)
Mathematical reasoning (max. 7)			
Pretest Mean (*SD*)	2.72 (1.63)	2.69 (1.58)	3.01 (1.81)
Posttest Mean (*SD*)	3.74 (1.56)	3.49 (1.72)	3.65 (1.69)
Gain score Mean (*SD*)	1.02 (1.86)	.79 (1.75)	.64 (1.83)
% Gain	14.6	11.3	9.1
Cohen’s *d* (95% CI)	.64 (.28–1.00)	.48 (.14–.83)	.37 (.02–.71)
Problem solving (max. 3)			
Pretest Mean (*SD*)	.71 (.73)	.69 (.71)	.83 (.75)
Posttest Mean (*SD*)	1.10 (.84)	1.07 (.91)	1.05 (.82)
Gain score Mean (*SD*)	.39 (.91)	.38 (1.06)	.21 (.91)
% Gain	13.0	12.7	7.0
Cohen’s *d* (95% CI)	.50 (.14–.85)	.47 (.12–.81)	.28 (−.06–.62)
